# Genomic structure and diversity of *Plasmodium falciparum* in Southeast Asia reveal recent parasite migration patterns

**DOI:** 10.1038/s41467-019-10121-3

**Published:** 2019-06-17

**Authors:** Amol C. Shetty, Christopher G. Jacob, Fang Huang, Yao Li, Sonia Agrawal, David L. Saunders, Chanthap Lon, Mark M. Fukuda, Pascal Ringwald, Elizabeth A. Ashley, Kay Thwe Han, Tin Maung Hlaing, Myaing M. Nyunt, Joana C. Silva, Kathleen E. Stewart, Christopher V. Plowe, Timothy D. O’Connor, Shannon Takala-Harrison, Artemisinin Resistance Confirmation, Characterization, and Containment (ARC3), Harald Noedl, Wasif A. Khan, Artemisinin Resistance Containment and Elimination (ARCE), Paul Newton, Myat P. Kyaw, Tracking Resistance to Artemisinin Collaboration (TRAC), Nicholas J. White, Arjen M. Dondorp, Nicholas P. Day, Charles J. Woodrow, Mehul Dhorda, M. Abul Faiz, Rick M. Fairhurst, Pharath Lim, Rupam Tripura, Mayfong Mayxay, Ye Htut, Francois Nosten, Aung Pyae Phyo, Sasithon Pukrittayakamee, Tran Tinh Hien, Nguyen Thanh Thuy Nhien, Olugbenga A. Mokuolu, Caterina I. Fanello, Marie A. Onyamboko

**Affiliations:** 1 Institute for Genome Sciences, University of Maryland School of Medicine, Baltimore, MD 21201 USA; 2 Graduate Program in Epidemiology and Human Genetics, University of Maryland School of Medicine, Baltimore, MD 21201 USA; 3 Wellcome Sanger Institute, Hinxton, CB10 1SA Cambridgeshire UK; 4 National Institute of Parasitic Diseases, Chinese Center for Disease Control and Prevention, Beijing, 102206 PR China; 5 Center for Geospatial Information Science, University of Maryland, College Park, MD 20742 USA; 6 Center for Vaccine Development and Global Health, University of Maryland School of Medicine, Baltimore, MD 21201 USA; 7 Armed Forces Research Institute of Medical Sciences, Bangkok, 10400 Thailand; 8 Armed Forces Research Institute of Medical Sciences, Khan Daun Penh, Phnom Penh, Cambodia; 9 Global Malaria Programme, World Health Organization, Geneva, 1202 Switzerland; 10 Mahidol-Oxford Tropical Medicine Research Unit (MORU), Faculty of Tropical Medicine, Mahidol University, Bangkok, 10400 Thailand; 11 Centre for Tropical Medicine and Global Health, Nuffield Department of Medicine, University of Oxford, Oxford, OX3 7FZ UK; 12 Department of Medical Research, Ministry of Health and Sports, Yangon, 11191 Myanmar; 13 Defence Services Medical Research Centre, Naypyitaw, Myanmar; 14 Duke Global Health Institute, Duke University, Durham, NC 27710 USA; 15 Department of Microbiology and Immunology, University of Maryland School of Medicine, Baltimore, MD 21201 USA; 16 Program in Personalized and Genomic Medicine, University of Maryland School of Medicine, Baltimore, MD 21201 USA; 17 Institute of Specific Prophylaxis and Tropical Medicine, Medical University of Vienna, Vienna, 1090 Austria; 18 International Centre for Diarrhoeal Disease Research, Bangladesh (icddr,b), Dhaka, 1212 Bangladesh; 19 Lao-Oxford-Mahosot Hospital-Wellcome Trust Research Unit (LOMWRU), Thanon Mahosot, Vientiane, Laos; 20 Asia Regional Centre & EQA Programme, Worldwide Antimalarial Resistance Network, Oxford, OX3 7FZ UK; 21 Malaria Research Group and Dev Care Foundation, Dhaka, Bangladesh; 22 Laboratory of Malaria and Vector Research, National Institute of Allergy and Infectious Diseases, National Institutes of Health, Rockville, MD 20852 USA; 23 Faculty of Postgraduate Studies, University of Health Sciences, Vientiane, 7322 Laos; 24 Shoklo Malaria Research Unit, Mahidol-Oxford Tropical Medicine Research Unit, Faculty of Tropical Medicine, Mahidol University, Mae Sot, Bangkok, 63110 Thailand; 25 Faculty of Tropical Medicine, Mahidol University, Bangkok, 10400 Thailand; 26 Centre for Tropical Medicine Oxford University Clinical Research Unit Vietnam (OUCRU), Quan 5, Ho Chi Minh City, Vietnam; 27 Department of Paediatrics and Child Health, University of Ilorin, Ilorin, 240 Nigeria

**Keywords:** Population genetics, Infectious-disease epidemiology, Parasite genomics, Malaria

## Abstract

Estimates of *Plasmodium falciparum* migration may inform strategies for malaria elimination. Here we elucidate fine-scale parasite population structure and infer recent migration across Southeast Asia using identity-by-descent (IBD) approaches based on genome-wide single nucleotide polymorphisms called in 1722 samples from 54 districts. IBD estimates are consistent with isolation-by-distance. We observe greater sharing of larger IBD segments between artemisinin-resistant parasites versus sensitive parasites, which is consistent with the recent spread of drug resistance. Our IBD analyses reveal actionable patterns, including isolated parasite populations, which may be prioritized for malaria elimination, as well as asymmetrical migration identifying potential sources and sinks of migrating parasites.

## Abstract (web summary)

Understanding genomic variation in *Plasmodium falciparum* parasites and inferring migration patterns can guide malaria elimination strategies. Using genome-wide data for 1722 parasites collected from 54 districts, the authors use identity-by-descent approaches to estimate regional parasite migration and spread of artemisinin drug resistance.

## Introduction

The emergence of *Plasmodium falciparum* resistance to both artemisinins and key partner drugs has compromised the efficacy of current first-line artemisinin-based combination therapies in the Greater Mekong Subregion, and poses a serious threat to renewed hopes for global malaria eradication^1^. To mitigate this threat, the World Health Organization has recommended elimination of *P. falciparum* malaria from the Greater Mekong Subregion by 2030^2^. As elimination efforts proceed, increasingly local information about malaria risk will be important for prioritizing resources and optimizing strategies for malaria elimination^3–5^. Estimates of parasite migration may be important in stratifying malaria risk; however, to be informative for elimination efforts, such estimates need to reflect recent patterns of parasite movement in time and space.

Genomic regions that have been inherited from a common ancestor are said to be identical-by-descent^6–8^, with the length of haplotypes shared between individuals being inversely proportional to the time since divergence from that common ancestor^9,10^. Shorter haplotypes, which have been broken down by recombination over time, indicate more historic demographic events, while longer haplotypes, which have undergone less recombination, are indicative of more recent events^11,12^. Identity-by-descent (IBD) approaches are increasingly used in human genomics for inference of recent demographic events^9,13^. Such approaches are now being used to document changes in malaria parasite population demography as a result of reduced malaria transmission^14^ or sweeping drug resistance mutations^15^ and to examine connectivity between populations^16^.

Patterns consistent with isolation-by-distance, a model predicting that populations that are closer in geographic distance are more genetically similar, have been shown using a variety of methods for many different organisms^17–19^. In the past, inferences about the relationship between genetic population structure and geographic distance often involved post hoc geographic interpretation of genetic subpopulations determined without consideration of their spatial coordinates^20,21^. However, more recently, approaches have been developed that explicitly model the spatial structure in the data^19,22,23^. One such approach is estimated effective migration surfaces (EEMS)^23^. The EEMS toolkit is a promising ecosystem of computational tools that couples geospatial mapping capabilities with gene-flow modeling to allow researchers to visualize geographic variation associated with genetic population structure. By finding contrasts between genetic distance and geographic distance, EEMS^23^ makes use of geo-referenced genomic data to visualize regions of relatively high- and low-effective migration within a geographic region, resulting in a visually intuitive representation of population structure within a given geographic area. In other words, for a given geographic distance between different sampling locations, EEMS estimates whether there is more or less migration compared to other locations of equal distance. In this study, we use estimates of IBD and EEMS to identify fine-scale population structure as well as migratory patterns of *P. falciparum* driven by contemporary and relatively recent demographic events.

## Results

### Genetic similarity and structure using IBD

**Fig. 1:**
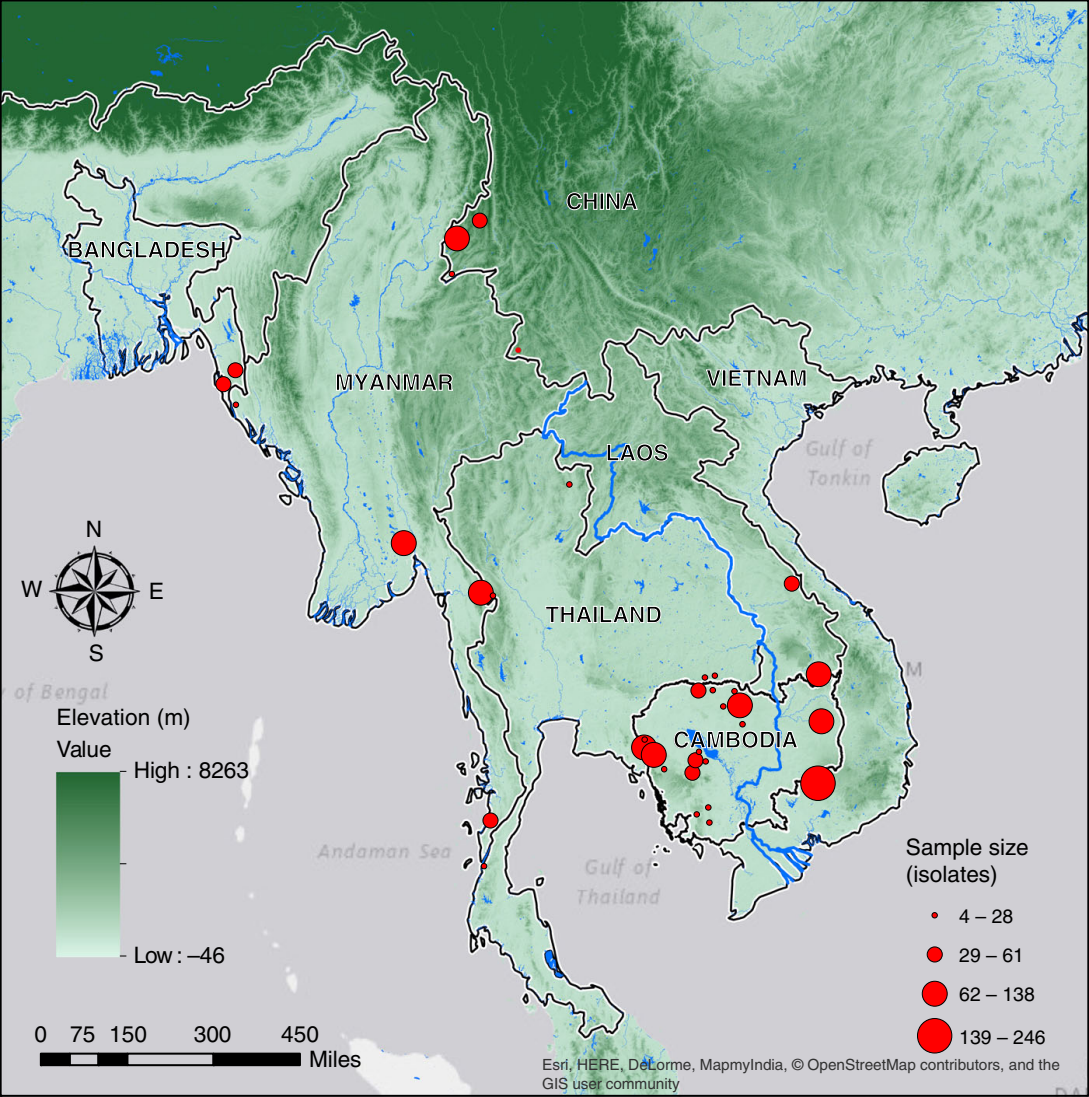
Sampling locations within the Greater Mekong Subregion and Bangladesh. Red circles mark districts where *P. falciparum* isolates were sampled. Circle area is proportional to the number of isolates sampled in that district. The SRTMGL3 elevation data was retrieved from the online EarthExplorer, courtesy of the NASA Land Processes Distributed Active Archive Center (LP DAAC), USGS/Earth Resources Observation and Science (EROS) Center, Sioux Falls, South Dakota, https://earthexplorer.usgs.gov/^65–68^

The dataset for this study consisted of 28,496 genome-wide single-nucleotide polymorphisms (SNPs) genotyped from 1722 clinical *P. falciparum* isolates collected from 54 districts within the Greater Mekong Subregion and Bangladesh during 2008–2013 (Fig. 1; Supplementary Table 1). We identified genomic segments that are IBD for each pair of isolates in the dataset. The IBD segments ranged in size from 0.5 to 225 cM of which segments smaller than 2 cM were excluded (Supplementary Fig. 1a)^24^. When aggregated over the entire parasite genome (~2500 cM)^25^ for each pair of isolates, the cumulative IBD sharing between sample pairs ranged from 0 to 1650 cM (Supplementary Fig. 1b).

**Fig. 2:**
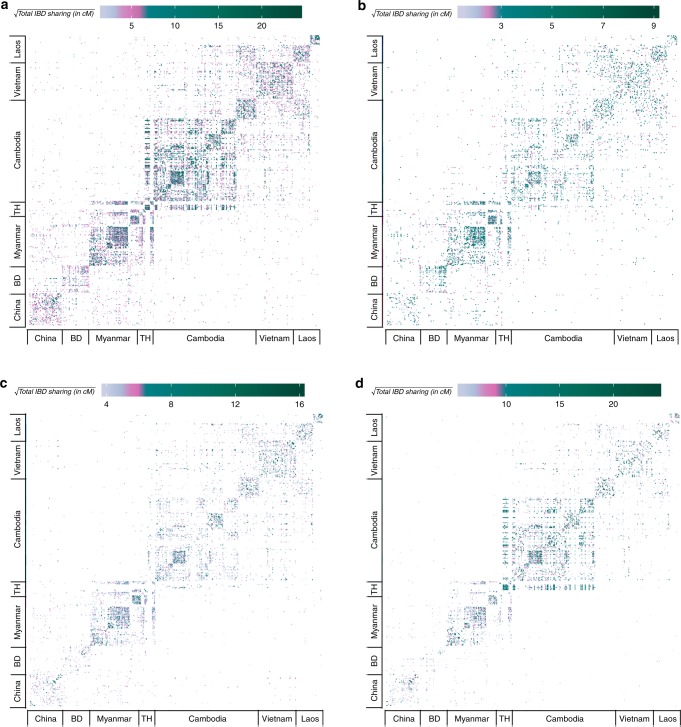
Cumulative pairwise IBD sharing between all parasites. **a** All segments greater than 2 cM, **b** 2–15 cM, **c** 15–30 cM, and **d** greater than 30 cM. Panels **b**–**d** are subsets of panel **a**. Each tile denotes the cumulative IBD sharing between isolates. Bangladesh and Thailand are abbreviated as BD and TH, respectively. The color indicates the magnitude of total IBD sharing

When examining pairs of isolates both within and between districts (Fig. 2), IBD was highest within individual countries. However, finer resolution at the district level revealed significant IBD sharing among districts in Cambodia, Laos, Myanmar, and Thailand (Fig. 2a, Supplementary Table 2). To determine the age of shared ancestry between isolates, we stratified the IBD estimates by the length of the segments, namely 2–15 cM, 15–30 cM, and greater than 30 cM, referring to distant, intermediate, and recent common ancestors, respectively. When focusing on the shorter segments (Fig. 2b, Supplementary Table 2), we observed larger estimates of IBD sharing between isolates within districts in Bangladesh and Myanmar and between districts along the Bangladesh–Myanmar border, implying that genetic similarity between these districts arose due to earlier migration events. Plots of IBD sharing based on segments of intermediate length (Fig. 2c, Supplementary Table 2) demonstrated more sharing between isolates within districts in China, Myanmar, Vietnam, and Laos, and between districts along the northern and southern Myanmar–Thailand border. IBD sharing based on the largest segments (Fig. 2d, Supplementary Table 2) was greatest between isolates within Cambodia and bordering regions of Thailand, indicating recent sharing between districts in these areas.

**Fig. 3:**
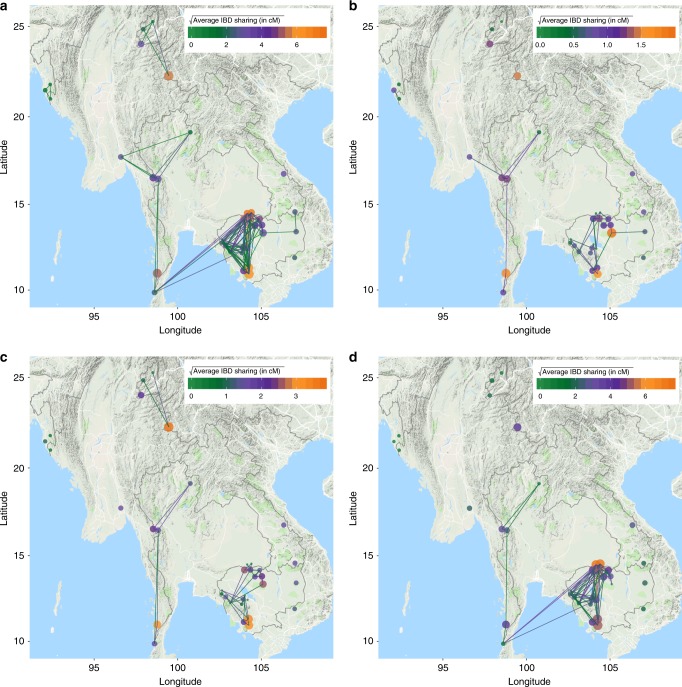
Regional relatedness within and between districts. **a** All segments greater than 2 cM, **b** 2–15 cM, **c** 15–30 cM, and **d** greater than 30 cM. Sharing of larger IBD segments indicates more recent migration. Circles represent the average IBD sharing within a district while lines represent the average IBD sharing between two districts. The color indicates the magnitude of IBD sharing while the area of the circle represents the average number of segments shared. Only district-pairs with >3% isolate-pairs demonstrating IBD sharing are included. Map data: Google, 2018

We next examined IBD sharing between district pairs, or regional relatedness. Regional relatedness was estimated as the average pairwise IBD sharing between isolates from two districts, stratified by IBD segment length (Fig. 3, Supplementary Fig. 2). Regional relatedness estimates based on short-IBD segments (Fig. 3b) were higher between districts along the Bangladesh–Myanmar border and along the Myanmar–Thailand border, but no connectivity was observed between districts in Cambodia and those along the Myanmar–Thailand border. Regional relatedness estimates based on IBD segments of intermediate length (Fig. 3c) continued to show connectivity between districts along the Myanmar–Thailand border and between districts in China, but again, no connectivity between the Myanmar–Thailand border and districts in Cambodia. However, some connectivity between districts in northern and central Cambodia and districts along the southern Myanmar–Thailand border was observed when examining regional relatedness based on the largest IBD segments. Within Cambodia (Supplementary Fig. 2), we observed little connectivity between the eastern and western districts based on regional relatedness estimates, regardless of stratification by IBD segment length. Regional relatedness between all districts in northern, central, and southern Cambodia increased over time, with the greatest connectivity observed between districts based on the largest IBD segments.

The presence of a greater number of genetically related parasites within a region is analogous to the presence of family structures in human populations. As is commonly done in population studies in humans, where genetically related individuals are excluded to avoid confounding, we performed analyses both including and excluding highly genetically related parasites (see Methods). The inclusion of genetically similar isolates increased the magnitude of regional relatedness estimates within some districts (Supplementary Table 1). However, overall patterns of IBD sharing were similar when highly genetically related parasites were included or excluded.

### Isolation-by-distance

The “*isolation-by-distance*” equilibrium model predicts that genetic differentiation increases with geographic distance, where significant deviations are explicable by admixture or isolation events^23,26,27^. However, large-scale migration events can potentially alter this equilibrium^12,28^. To test isolation-by-distance in this dataset, we compared the average pairwise IBD sharing between districts to their geographic distance using a Mantel test. As expected, we observed a negative correlation (Spearman *R* = −0.4263; *p* value < 1 × 10^−4^; 10,000 permutations) between regional relatedness and geographic distance (Supplementary Fig. 3). This finding was consistent when stratifying by IBD segment length. Even though the isolation-by-distance model fit for most districts, we observed some subtle deviations from this expectation, such as Bago (near Yangon) and Kawthaung (southern border) in Myanmar, that showed higher estimates of regional relatedness with geographically distant districts than proximal ones. Also, within Cambodia, we observed higher estimates of regional relatedness with geographically distant districts than proximal ones for districts such as Samlout (western Cambodia) and Phnom Sruoch (southern Cambodia).

### Directional migration of *P. falciparum*

**Fig. 4:**
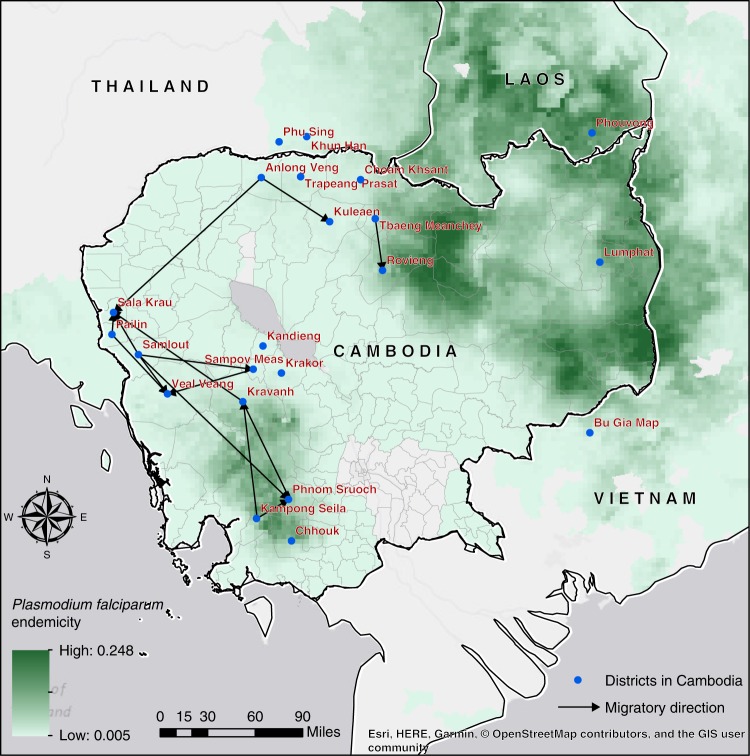
Asymmetrical migration between districts in Cambodia. Directional migration was inferred using IBD estimates between nonadmixed and admixed isolates across districts. Statistically significant asymmetrical migration between two districts is indicated by an arrow. Malaria endemicity is indicated in green. Map data: Malaria Atlas Project (https://map.ox.ac.uk/)^69^

To estimate asymmetrical migration between districts we utilized a relatively simple method based on IBD estimates after stratifying isolates into nonadmixed and admixed groups (Supplementary Methods, Supplementary Figs. 4 and 5). Under the premise that nonadmixed isolates represent the native population within the region while admixed isolates represent a demographic admixture event between the isolates from two regions, the regional relatedness estimates based on the nonadmixed isolates from one district (*D1*) and the admixed isolates of a second district (*D2*) provide insights into the possibility of migration from *D1* to *D2*. As seen in Supplementary Table 3, we observed significant asymmetrical migration estimates based on permutation tests (*p* value < 5 × 10^−4^) from Bago (central Myanmar) to Ranong (southern Thailand) and from Kawthaung (southern Myanmar) to Ranong (southern Thailand). We also detected several asymmetrical migrations between districts in central, western, northern, and southern Cambodia (Fig. 4). In western Cambodia, Samlout may represent a source of migrating parasites, with three instances of asymmetrical migration out of this district, while Sala Krau in the west and Phnom Sruoch in the south may be sinks with three instances of asymmetrical migration into these districts (Fig. 4).

### Shared IBD haplotypes in drug-resistant parasites

To further elucidate the spread of artemisinin-resistant parasites, we repeated the regional relatedness analysis using samples from Cambodia and its neighboring provinces with parasite isolates possessing an artemisinin resistance mutation in the propeller region of the *kelch13* gene (K13) (*N* = 475) and those isolates possessing the wild-type allele (*N* = 437). Average IBD sharing estimates were higher within districts than between districts for both K13 mutant and K13 wild-type isolates (Table 1). We also observed higher average IBD sharing estimates for K13 mutant isolates compared to K13 wild-type isolates. This sharing represented genome-wide IBD sharing and not sharing exclusively in genomic regions harboring drug resistance alleles. Overall, within districts in Cambodia and neighboring regions, there was 2.45-fold greater total IBD sharing between K13 mutant isolates (average IBD_within_ = 17.31 cM) than between K13 wild-type isolates (average IBD_within_ = 7.07 cM). Between districts, there was 5.88-fold greater IBD sharing between K13 mutant isolates (average IBD_between_ = 5.59 cM) than between K13 wild-type isolates (average IBD_between_ = 0.95 cM). These differences were statistically significant, based on permutation tests (*p* value < 1 × 10^−5^ for both comparisons). When stratified by IBD segment length, the significant difference in regional relatedness of K13 mutant and K13 wild-type isolates was only recapitulated at segment lengths greater than 30 cM (Table 1), showing the ability of this approach to capture the spread of artemisinin resistance both within and between districts that is known to have occurred rapidly in the recent past.

**Table 1:** Average IBD sharing among parasites with and without artemisinin resistance mutations

IBD segment length	K13 mutant^a^	K13 wildtype^a^	*p* value^b^	K13 mutant^c^	K13 wildtype^c^	*p* value^b^
>2 cM	17.31	7.07	<10^−5^	5.59	0.95	<10^−5^
2–15 cM	0.67	0.54	0.057	0.21	0.12	0.478
15–30 cM	2.59	2.1	0.17	0.62	0.31	0.402
>30 cM	14.06	4.43	<10^−^^5^	4.76	0.52	<10^−5^

^a^Average IBD sharing (cM) within districts

^b^*p* values were computed using a permutation test with 100,000 permutations

^c^Average IBD sharing (cM) between districts

Regional relatedness of K13 mutant isolates based on short-IBD tract lengths between 2 and 15 cM showed large average within-district and between-district IBD estimates for all three districts in southern Cambodia, namely Chhouk, Kampong Seila, and Phnom Sruoch (Supplementary Fig. 6). Focusing on intermediate IBD tract lengths, we continued to observe increased IBD sharing among K13 mutant isolates within and between the southern Cambodian districts. In addition, we observed increased average IBD estimates within Sampov Meas in central Cambodia and Bu Gia Map in Vietnam. Lastly, regional relatedness estimates among K13 mutant isolates based on segment lengths greater than 30 cM, showed increased IBD sharing estimates within Phu Sing along the Cambodia–Thailand border and also high average IBD sharing between Phu Sing and districts in northern, central, and western Cambodia that are geographically distant districts.

### Migratory patterns of *P. falciparum* based on EEMS

**Fig. 5:**
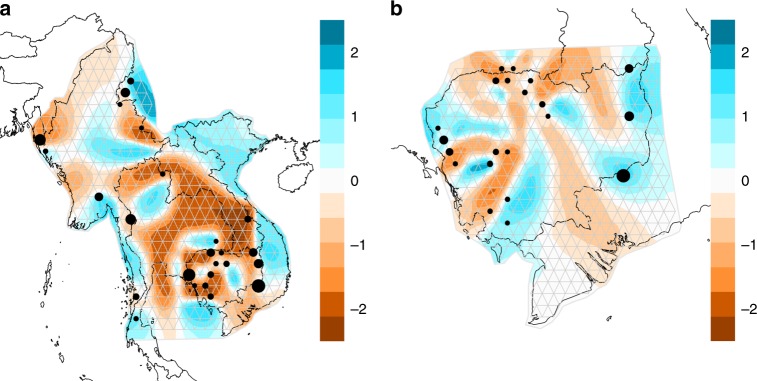
EEMS contours illustrating relative migration. Each circle is a deme consisting of one or more districts, with area proportional to the number of isolates in the deme. Panels depict relative parasite migration in **a** Southeast Asia and **b** Cambodia, with brown contours indicating lower levels of migration and blue contours indicating higher levels of migration

Estimated effective migration surface (EEMS) contours^23^ illustrate the relative high or low effective migration within the Greater Mekong Subregion (Fig. 5a) and within Cambodia and its neighboring regions (Fig. 5b). Using this approach, we detected potential barriers to migration across central Thailand (where there is no malaria) and along some political borders (e.g., the China–Myanmar border, western Myanmar border, and the northern Thailand–Myanmar border). These potential barriers also showed high-posterior probabilities > 0.90 (Supplementary Fig. 7) in the Bayesian estimation of migration parameters. Within Cambodia, there were potential barriers to migration separating the eastern and western parts of the country, as well as within some of the northern and western districts. The barriers in the northern and western districts showed the highest posterior probabilities (>0.90) of migration parameters, while the barrier in central Cambodia (where sampling was limited), was not supported by a posterior probability >0.90 (Supplementary Fig. 7).

To test the robustness of the EEMS model, we undertook a population-level jackknife sampling approach to iteratively exclude isolates from a single district and estimate migratory patterns. We observed similar patterns of migratory surfaces with a few exceptions (Supplementary Figs. 8 and 9). The exclusion of isolates from Ruili, China, changed the migratory patterns along the China–Myanmar border (Supplementary Fig. 8). Overall, EEMS recapitulated some patterns of migration detected by regional relatedness analyses using short IBD tracts. Both analyses detected increased relatedness and decreased migration barriers between districts along the northern and southern borders for Myanmar and Thailand, and the southern provinces of Cambodia.

## Discussion

Modeling *P. falciparum* population structure and migration patterns can provide actionable evidence to help national malaria control programs prioritize resources and plan efficient strategies for malaria elimination by identifying where parasites are moving, or barriers to parasite movement. IBD-based approaches can elucidate fine-scale population structure and patterns of parasite migration in the Greater Mekong Subregion, a priority region for malaria elimination owing to the emergence and spread of artemisinin-resistant *P. falciparum* there. Pairwise IBD sharing revealed not only increased sharing between isolates within a country, but also fine-scale population structure at the district level within some countries. This more local estimation of parasite migration patterns is ideal for informing elimination strategies but has been challenging to detect with other methods such as ADMIXTURE (Supplementary Fig. 4), principal component analysis (Supplementary Fig. 5), or measures of genetic differentiation^16^. Average IBD sharing estimates between geographic locations provided insights into the spread of parasite haplotypes within and between countries. We observed both high levels of IBD between districts in close proximity within countries and near political borders and also between geographically distant districts. These more distant migrations could be driven by human movement, geographic topography, and/or sweeping artemisinin resistance. Using IBD tract length as a surrogate for time, we found patterns consistent with the spread of *P. falciparum* in the distant past along the northern and southern Myanmar–Thailand border, and a more recent relatively rapid spread of parasite haplotypes that mirrors the contemporary spread of artemisinin resistance. IBD estimates also facilitated determination of the direction of migratory patterns between northern, western, central, and southern districts in Cambodia, allowing identification of potential sources and sinks of migrating parasites.

Several studies have examined the population structure of *P. falciparum* in parts of Asia using different approaches^16,29–43^. In this study, we show potential barriers to migration between eastern and western Thailand and between eastern and western Cambodia. These findings are consistent with studies that have shown population differences between western, eastern, and southern Thailand^34^, as well as studies of drug resistance and population structure within Cambodia that have shown less drug resistance and different parasite subpopulations in the east compared to the west^37,44^. We also observed recent increased IBD sharing between districts in Cambodia and along the Thailand–Cambodia border, consistent with the findings of Cerqueira et al.^15^ who observed increased IBD sharing over time in western Thailand. These authors hypothesized that this increase in IBD sharing in recent years was due to a combination of reduced malaria transmission in the area, as well as the spread of drug-resistant lineages. These factors are likely also contributing to the increased IBD observed over time in western, northern, and southern Cambodia where artemisinin and partner drug resistance have become well-established^43,45^ and where elimination efforts have been initially focused. This scenario is further supported by our observation that parasites with artemisinin-resistance mutations show greater IBD sharing between districts compared to wild-type parasites, when examining the largest IBD fragments. We also observed low connectivity between parasites from the China–Myanmar border and those in other regions of the Greater Mekong Subregion, consistent with the findings of Wei and colleagues who showed distinct population structure between parasites in Yunnan province and those in southernmost Hainan^42^. However, sampling of parasites in regions outside of Cambodia was limited and precluded more fine-scale evaluations of connectivity in regions of Myanmar and bordering countries.

Sampling of parasites was not spatially uniform across geographic locations, which could create some spatial uncertainty in areas with less sampling. In addition, the EEMS approach assumes that migration occurs contiguously between uniformly spaced demes^23^. Such assumptions may not be valid if infected individuals migrate over large distances and when malaria transmission is heterogeneous throughout the landscape. Recent EEMS publications^23,46^ and our own use of a leave-one-out approach^47^ illustrated in Supplementary Figs. 8 and 9, suggest that migration patterns estimated using this method are relatively robust to irregular sampling, with only minor movement of the borders of migration barriers or regions of increased gene flow. However, the posterior probabilities supporting the observed patterns tend to be greater in areas with more sampling (Supplementary Fig. 7), suggesting some uncertainty in areas with limited or no samples contributing to the analysis. In this case, we had more dense sampling in regions of Cambodia with emerging artemisinin resistance^44,48,49^, providing increased resolution in migration estimates based on EEMS in that region. The overall migration patterns observed are similar at both regional and more local scales, with the major migration barrier between western and eastern Cambodia identified in both analyses. This approach is similar to the global EEMS assessment of human migration recently published by Peter et al.^46^. where some global conclusions were drawn followed by specific assessments and analysis of more refined regions of dense sampling, such as Europe. By modeling EEMS at different scales we delineate specific migration features within Cambodia, while contextualizing with regard to regional migration patterns. In addition to more sampling in regions of the Greater Mekong Subregion outside of Cambodia, ongoing work is focused on making EEMS more spatially explicit and on further understanding how irregular sampling, both in terms of geographic location and sample size, affect EEMS model outcomes and posterior probabilities.

Also, while both IBD sharing and EEMS demonstrate some similar migration patterns, particularly when examining IBD segments of small and intermediate lengths, EEMS may not be capturing more recent patterns inferred by examining larger shared IBD segments^50^. For example, when larger IBD segments are examined, we observed a large degree of regional relatedness within Cambodia, while EEMS indicates several barriers to migration within Cambodia. Ongoing work aims to modify the distance metric used in the EEMS analysis to include distances more reflective of recent demography, including distances based on IBD or rare variation. Such modifications should allow EEMS visualizations to represent more recent migration patterns.

Based on IBD, we are able to observe population structure at the district level. Microstratification of malaria risk to more local levels (e.g., villages) would be desirable for public health officials who are planning and carrying out interventions. As a next step, we will apply these approaches to determine whether population structure can be detected at the village level. A recent study by Taylor et al.^16^ used IBD to show isolation-by-distance over geographic distances less than 100 km along the Thailand–Myanmar border, suggesting that more local stratification of *P. falciparum* migration using this approach is possible. In addition, migration patterns can be used to infer the direction of parasite movement and identify sources and sinks of parasite transmission. Our analyses point to multiple directions of *P. falciparum* migration not only between proximal districts in northern, southern, and western parts of Cambodia but also between geographically distant districts across central, northern, and western Cambodia. Future studies will focus on validation of these findings based on estimates of human mobility within the region and finer mapping of parasite population structure and migration at the local level. If validated, these approaches may allow strategic targeting of interventions for elimination^3–5^. For example, elimination programs might first target more isolated parasite populations with low migration and low parasite diversity (e.g., the China–Myanmar border, in this study), as these areas are less likely to experience reintroduction of parasites from other areas. Likewise, elimination programs may want to target sources of transmission prior to sinks, again, to avoid reintroduction of *P. falciparum* from the former to the latter. Based on EEMS results, the landscape may be divided into areas of relatively higher or lower parasite migration, which may allow identification of discrete geographic regions separated by barriers to migration that could be exploited when planning intervention strategies. These approaches thus hold promise for accelerating the pace of malaria elimination in this region and preventing the global spread of multidrug resistance.

## Methods

### Sample collection and parasite genotyping

Samples were collected from completed studies involving passive surveillance for clinical malaria and clinical trials of *P. falciparum* infections confirmed by microscopy or polymerase chain reaction^44,48,49,51–56^. The study sites spanned the Greater Mekong Subregion (comprised of Cambodia, Laos, Myanmar, Thailand, Vietnam, and the Yunnan Province of China), bordering regions of Bangladesh, and two sites in Africa. All samples were collected from 2008 to 2013 with informed consent from symptomatic individuals that met the inclusion criteria of the initial study protocol with prior approval from the local ethical review boards^44,48,49,51–56^. Parasite genotyping and genomic analyses were undertaken after prior approval of the University of Maryland School of Medicine Institutional Review Board.

A total of 2185 samples were genotyped. Single-nucleotide polymorphisms (SNPs) were either called from whole-genome sequences generated at the Wellcome Sanger Institute as part of the MalariaGEN *Plasmodium falciparum* Community Project (*n* = 1468)^57^, or, for samples that did not meet quality control criteria for whole-genome sequencing or were not part of the Community Project (*n* = 717), were genotyped using a *P. falciparum*-specific NimbleGen 4.2M probe custom DNA microarray^58^. The same nucleotide positions typed on the microarray were extracted from whole-genome data for analysis. Genotyping data are publicly available through the MalariaGEN website (https://www.malariagen.net/data/p-falciparum-community-project-jan-2016-data-release) or through the NIH Gene Expression Omnibus (www.ncbi.nlm.gov/geo/) (Accession number: GSE100704) and European Variation Archive (Accession number: PRJEB28530). Based on previous analyses, 28,496 SNPs could be reliably typed^58^. Beagle software^59^ was used to impute missing SNP calls for genotyped isolates. ADMIXTURE^60^ was used to cluster parasites prior to imputation. Parasites with 100% ancestry values in each cluster were imputed within that cluster, and these imputed pures were used as a reference set to impute admixed parasites. A genotype probability of 90% was used to call an imputed SNP. Only biallelic SNPs were included in the dataset. Heterozygous SNP calls indicative of the presence of multiple parasite clones were coded as missing and thus not included in the analysis of haplotypes. Samples and SNPs were excluded after applying missingness cut-offs of 10% for samples and 15% for SNPs using the PLINK genomic analysis toolkit (version 1.9)^7,61^. District-level geographic coordinates were available for 1722 samples that were used for downstream analysis.

### IBD estimation

The SNPs genotyped and identified across all samples were separated into individual chromosomes. Only bi-allelic SNPs were retained for estimation of IBD measures after excluding all singletons. The final set of bi-allelic SNPs were utilized to compare all samples to identify genomic segments that are IBD using the BEAGLE software tool (version 4.1)^24,62^. Genetic map files utilized to identify IBD segments and convert chromosomal positions (bp) to genetic positions (cM) were determined from supplementary information provided in Jiang et al.^25^. IBD tracts of lengths 2 cM or longer were inferred from the BEAGLE results. Since BEAGLE assesses IBD tracts for each haplotype of a diploid genome, we additionally merged duplicate and overlapping IBD tracts within each pair of samples generated due to the haploid nature of the *P. falciparum* genome to generate a final set of nonduplicated, nonoverlapping IBD segments of length 2 cM or longer shared between two samples. Furthermore, cumulative IBD sharing between two samples was utilized to identify genetically related samples. Two samples were considered highly genetically similar if greater than 625 cM (25%) of the genome was identical-by-descent (i.e., reflecting two meiotic events). Samples were then identified iteratively until no two samples shared greater than 625 cM in cumulative IBD tract lengths.

### Genetic similarity measures using IBD estimates

We computed the cumulative shared IBD estimates, the cumulative number of shared IBD tracts, and the average IBD tract length for every pair of individuals. We also stratified the IBD tracts by length into three bins of IBD lengths between 2 and 15 cM, 15 and 30 cM, and greater than 30 cM based on the distribution of lengths observed in Supplementary Fig. 1a resulting in four IBD sharing matrices for each pair of individuals. The bins of IBD length were chosen based on the distribution of IBD segment lengths (shown in Supplementary Fig. 1). The highest frequency was observed for IBD segments with lengths between 10 and 30 cM. Hence, we divided the IBD segments into the three bins, namely 2–15 cM (~16% of all segments), 15–30 cM (~28% of all segments), and greater than 30 cM (~56% of all segments). To assess genetic similarity between geographic locations, the samples were assigned the geographic location inferred from the collection site of the sample. Pairwise IBD sharing between two regions *D1* and *D2* (regional relatedness) were computed from the mean length of total IBD sharing for all pairs of individuals where one individual is from *D1* and the other individual is from *D2*. Total number of pairs, *N*_Pairs_ = *ND1* × *N*_*D2*_ if *D1* ≠ *D2* and *N*_Pairs_ = *N* × (*N* − 1)/2 if *D1* = *D2*. Regional relatedness estimates can be noisy for regions with small sample sizes, hence districts with fewer than three isolates were excluded. Regional relatedness was estimated including and excluding genetically similar isolates to illustrate regional relatedness driven by genetically similar isolates. Regional relatedness was also estimated based on stratification of IBD segments based on segment length. In addition, these estimates were further stratified by nonadmixed and admixed samples within a population and alternatively were estimated specifically for drug-sensitive and drug-resistant parasites in a population. To test for significance in the latter, we compared the average total IBD sharing between K13 mutant and K13 wild-type isolates within and between districts and computed significance by permuting across the mutation status. These measures of genetic similarity between geographical regions have been used for visualization and downstream computational analyses. Visualizations were generated using R packages ggplot2^63^, ggmap^64^, and geographical coordinates for each district.

### Genetic similarity compared to isolation-by-distance

Using longitude and latitude coordinates for each of the districts, we calculated the geographical distance between two populations. The geographical distance matrices of distance measures between each population pair were compared to the genetic similarity matrices computed from IBD sharing estimates. To quantify the correlation between the geographical distance and genetic distance matrices, we use the Mantel test (provided in the R package “ecodist”). The Mantel test was computed using 10,000 permutations to generate a significance *p* value estimate. In addition, to model the expected decay of IBD sharing with increasing geographical distance, we correlated the genetic similarity and geographical distance vectors of a single population with every other population using the Spearman method to compute correlation *R* values and significance *p* values.

### Estimated effective migration surfaces

Estimated effective migration surfaces (EEMS) is a new approach to estimate genetic migration patterns over a given geographic region^23^. The EEMS analysis involves covering the study area with a dense equidistant triangular grid (Voronoi network), and each sample location is adjusted to the closest deme (vertex) on the grid. A Bayesian approach is then used to estimate migration parameters based on a stepping-stone model, which assumes individuals migrate locally between demes and each deme exchanges migrants only with its neighbors. We computed genetic dissimilarity matrices using EEMS and assigned geographical coordinates to each sample from each district to contrast geographic and genetic distances between demes. Migration surface contours were estimated using 400 demes for all Greater Mekong Subregion districts as well as for a subset of isolates from Cambodia and neighboring districts. The MCMC analysis was run for 15,000,000 MCMC iterations including 14,000,000 burn-in iterations and repeated using 10 different seeds to ensure the convergence of the MCMC chains as well as the accepted distributions of parameters as specified by the developers. Final spatial visualizations illustrating migratory surfaces were generated using R scripts provided by EEMS. In addition, to test the robustness of the models, we applied a jack-knife sampling approach and repeated the EEMS runs after iteratively excluding isolates from a single district.

### Migratory patterns using IBD estimates

To provide insights into the migratory patterns of *P. falciparum* within the Greater Mekong Subregion, we introduced a relatively simple method for detection of asymmetric migration using IBD estimates. We stratified the samples from a single geographical location into nonadmixed and admixed samples using admixture estimates from a prior analysis. We computed average estimates of IBD sharing (IBD_*D1*,*N-D2*,*N*_) between the nonadmixed samples of *D1* and the nonadmixed samples of *D2* and compared them to the IBD estimates between the nonadmixed samples from one population and the admixed samples from the other population (IBD_*D1*,*N-D2*,*A*_) and the converse (IBD_*D2*,*N-D1*,*A*_). Hence, we computed the regional relatedness between nonadmixed isolates (*N*) from one district to the admixed isolates (*A*) of another district (RR_*D1*,*N-D2*,*A*_) and vice versa (RR_*D2*,*N-D1*,*A*_) for all district pairs. We also computed the regional relatedness between the nonadmixed isolates from both districts (RR_*D1*,*N-D2*,*N*_), which indicates the potential of the isolates of the two districts to share a recent common ancestor. Only district pairs where the regional relatedness estimate RR_*XN-YA*_ or RR_*YN-XA*_ was greater than RR_*D1*,*N-D2*,*N*_ were considered. These genetic similarity measures between the nonadmixed and admixed samples across populations were used to estimate the relative levels of migration between the two populations. The significance of the difference in relative migration levels was estimated using permutation tests.

### Disclaimer

P.R. is a staff member of the World Health Organization. P.R. alone is responsible for the views expressed in this publication and they do not necessarily represent the decisions, policy or views of the World Health Organization. The views expressed are those of the authors and do not reflect those of the U.S. Department of Defense or the U.S. Government. 

### Reporting summary

Further information on research design is available in the Nature Research Reporting Summary linked to this article.

## Supplementary information

Supplementary Information

Reporting Summary

## Data Availability

Genotyping data are publicly available through the MalariaGEN website ([https://www.malariagen.net/data/p-falciparum-community-project-jan-2016-data-release]) or through the NIH Gene Expression Omnibus (Accession number GSE100704) and European Variation Archive (Accession number PRJEB28530).
