# Supplementary material for: Genomic structure and diversity of Plasmodium falciparum in Southeast Asia reveal recent parasite migration patterns
Source: Nat Commun. 2019 Jun 17;10:2665. doi: 10.1038/s41467-019-10121-3 (PMC6572796; doi:10.1038/s41467-019-10121-3)
Supplement: Supplementary file 1 — Supplementary Information [file 41467_2019_10121_MOESM1_ESM.pdf]

Supplementary Information

Genomic structure and diversity of *Plasmodium falciparum* in Southeast Asia reveal recent parasite migration patterns

Shetty *et al.*

Supplementary Methods

After excluding ~636 samples considered to be genetically similar specifically for admixture analysis, SNPs were pruned based on linkage disequilibrium using the PLINK genomic analysis toolkit (version 1.9)[1,2]. SNPs were assessed in a window size of 50 SNPs with a step size of 10 SNPs and a pairwise r^2 threshold of 0.1. The final set of ~10,000 SNPs was used as input for the ADMIXTURE software (version 1.3)[3,4] to determine ancestral population proportions in each sample. Admixture estimates were computed using the parameters specified for haploid chromosomes over a varying number of clusters (K) ranging from 1 to 40 with 10 technical replicates each. A best value for K was assessed by maximizing the log-likelihood across replicates for a single K value and minimizing the cross-validation (CV) error between different K values. The cluster proportions for each sample determined by the best value of K were plotted using R statistical programming after grouping them by their geographical locations. The samples were also stratified into non-admixed and admixed groups using an admixture proportion cut-off of 70% belonging to a single ancestral population. The samples were assigned the geographic location as inferred from the collection site of the sample.

We further analyzed the data to infer preliminary population structure using Principal Components Analysis (PCA) as implemented by the KING software (version 1.4)[5]. Additional outliers (three isolates each from Cambodia and Myanmar) based on their clustering with isolates from Africa were identified and excluded from downstream analyses. Analysis was repeated for a subset of samples originating from districts in Cambodia and neighboring provinces in Laos, Thailand, and Vietnam. The first and second principal components were plotted using the R statistical program illustrating geographical regions in distinct colors.

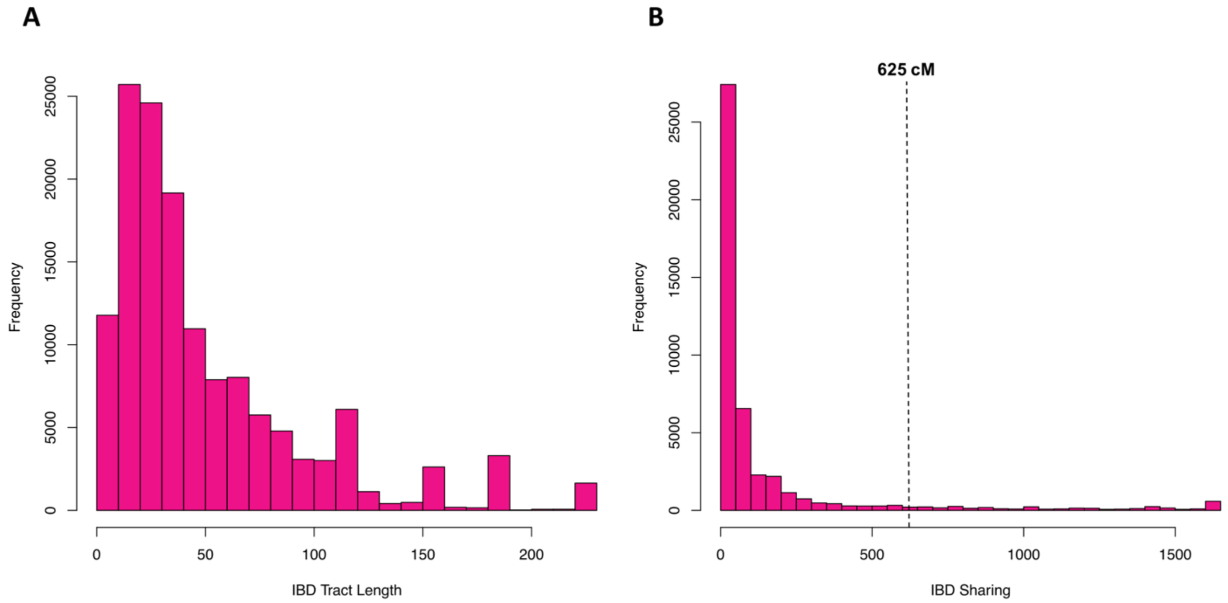

Supplementary Figure 1. Distribution of IBD tract lengths estimated using BEAGLE. (A) Histogram of individual IBD tract lengths, (B) Histogram of total IBD sharing after aggregating the IBD tracts for each sample pair based on 32,675 (2.2%) isolate pairs that showed non-zero IBD sharing including 2,850 isolate pairs with IBD sharing > 625cM.

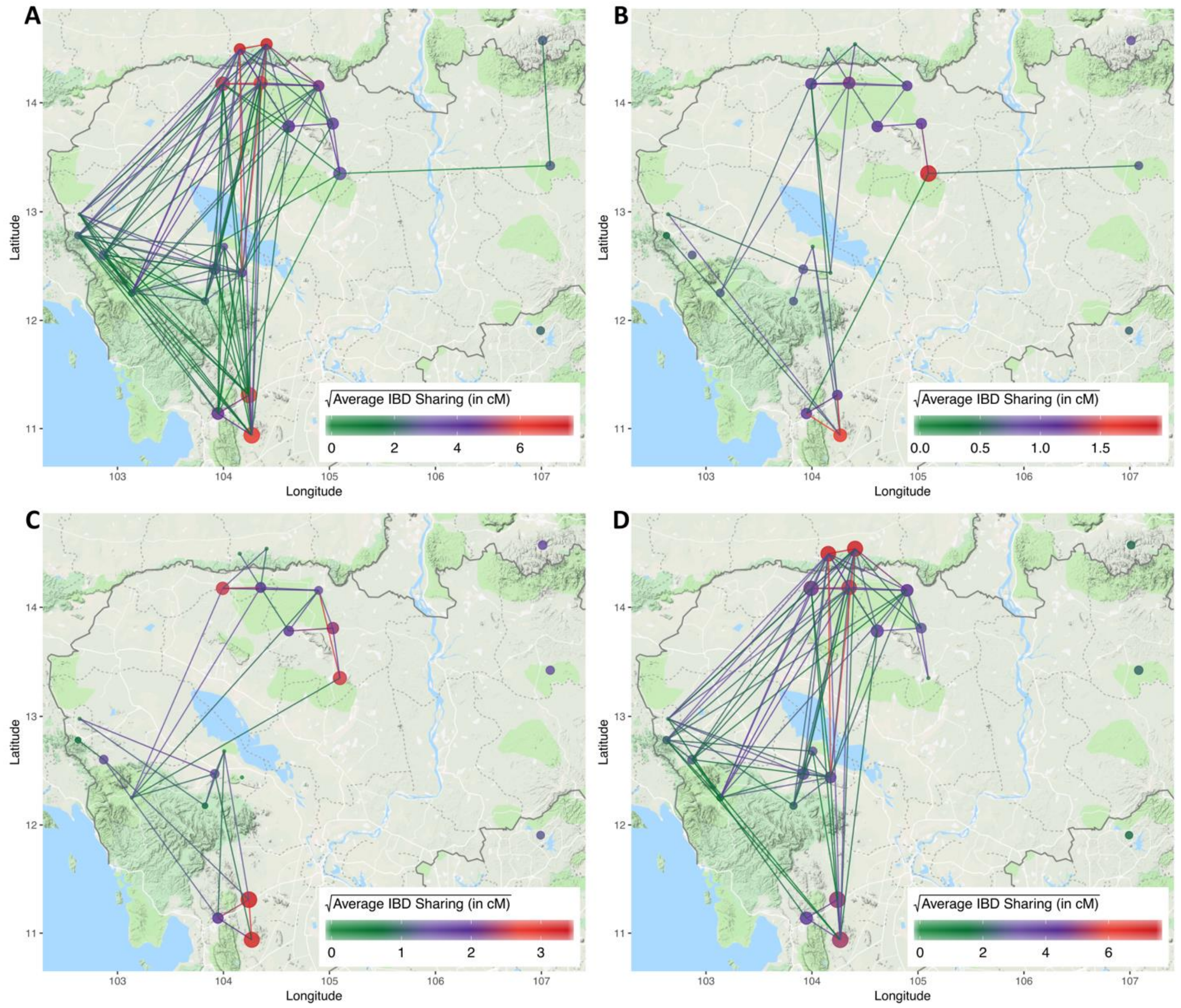

Supplementary Figure 2. Regional relatedness within and between Cambodian districts. (A) All segments greater than 2cM, (B) 2cM – 15cM, (C) 15cM – 30cM, and (D) greater than 30cM. Sharing of larger IBD segments indicates migration that is more recent. Circles represent the average IBD sharing within a district while lines represent the average IBD sharing between two districts. The color indicates the magnitude of IBD sharing while the area of the circle represents the average number of segments shared. Only district-pairs with >3% isolate-pairs demonstrating IBD sharing are included. Map data: Google, 2018.

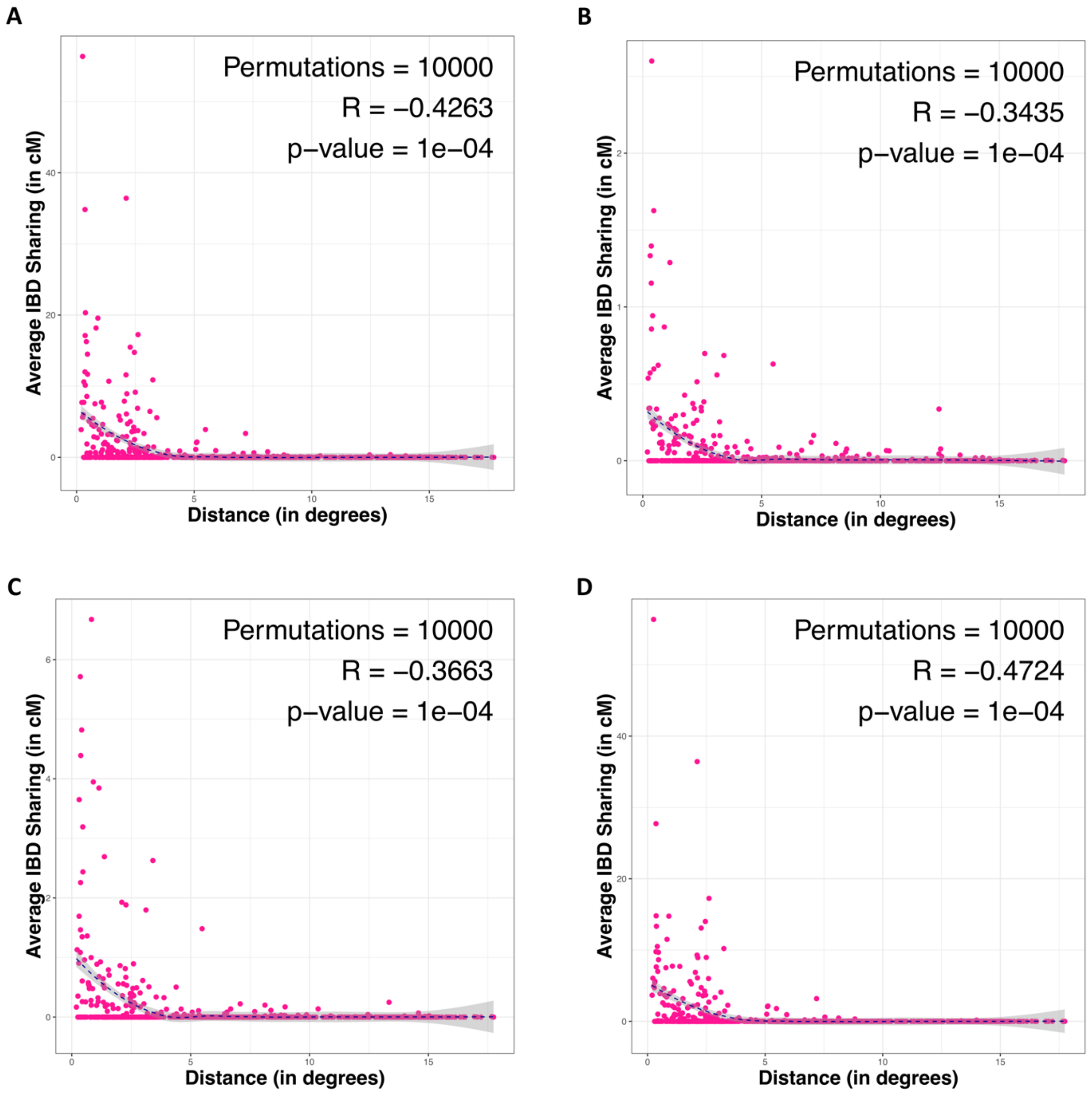

Supplementary Figure 3. Isolation-by-distance. Decay of average IBD (shown in logarithmic scale) as a function of distance stratified by IBD segment length; greater than 2cM (A), 2cM – 15cM (B), 15cM – 30cM (C) and greater than 30cM (D). R indicates Spearman correlation values.

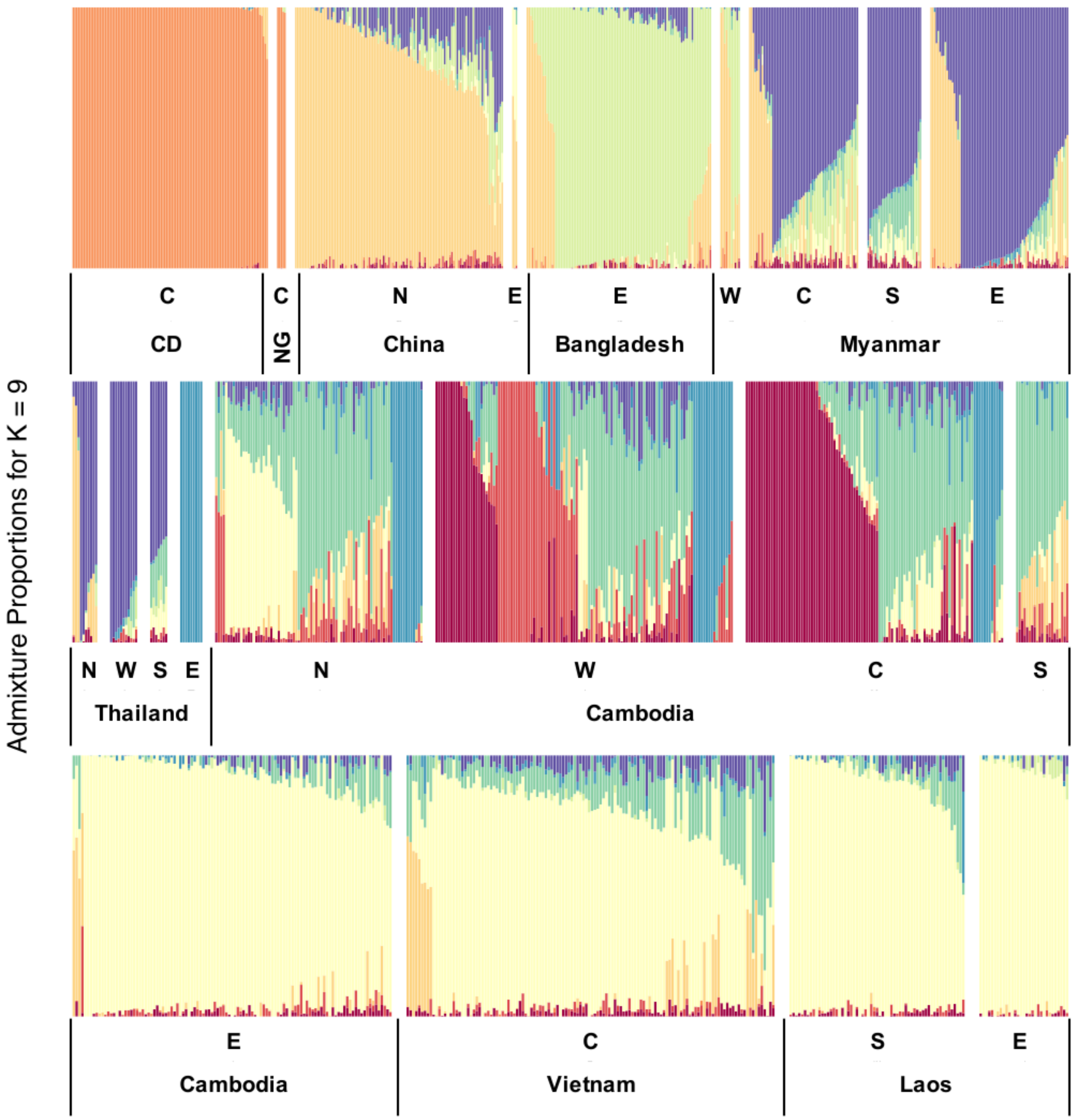

Supplementary Figure 4. Admixture analysis. ADMIXTURE was used to illustrate the proportion of ancestral populations (K) for each sample. For K=9, each vertical line represents a single sample, with color denoting the ancestral proportion in that sample. N, W, C, S, and E denote northern, western, central, southern, and eastern provinces within a country. Democratic Republic of Congo and Nigeria are abbreviated as CD and NG, respectively. We found nine ancestral clusters, with one cluster comprised of samples from Africa. One cluster was found predominantly in isolates from Eastern Cambodia, Vietnam, and Laos. Isolates from China and Bangladesh showed low levels of admixture representing two distinct clusters, while isolates from Myanmar and Thailand represented a third distinct cluster. Within several Cambodian populations (except the eastern districts), we observed both non-admixed and highly admixed isolates representing the remaining four ancestral clusters.

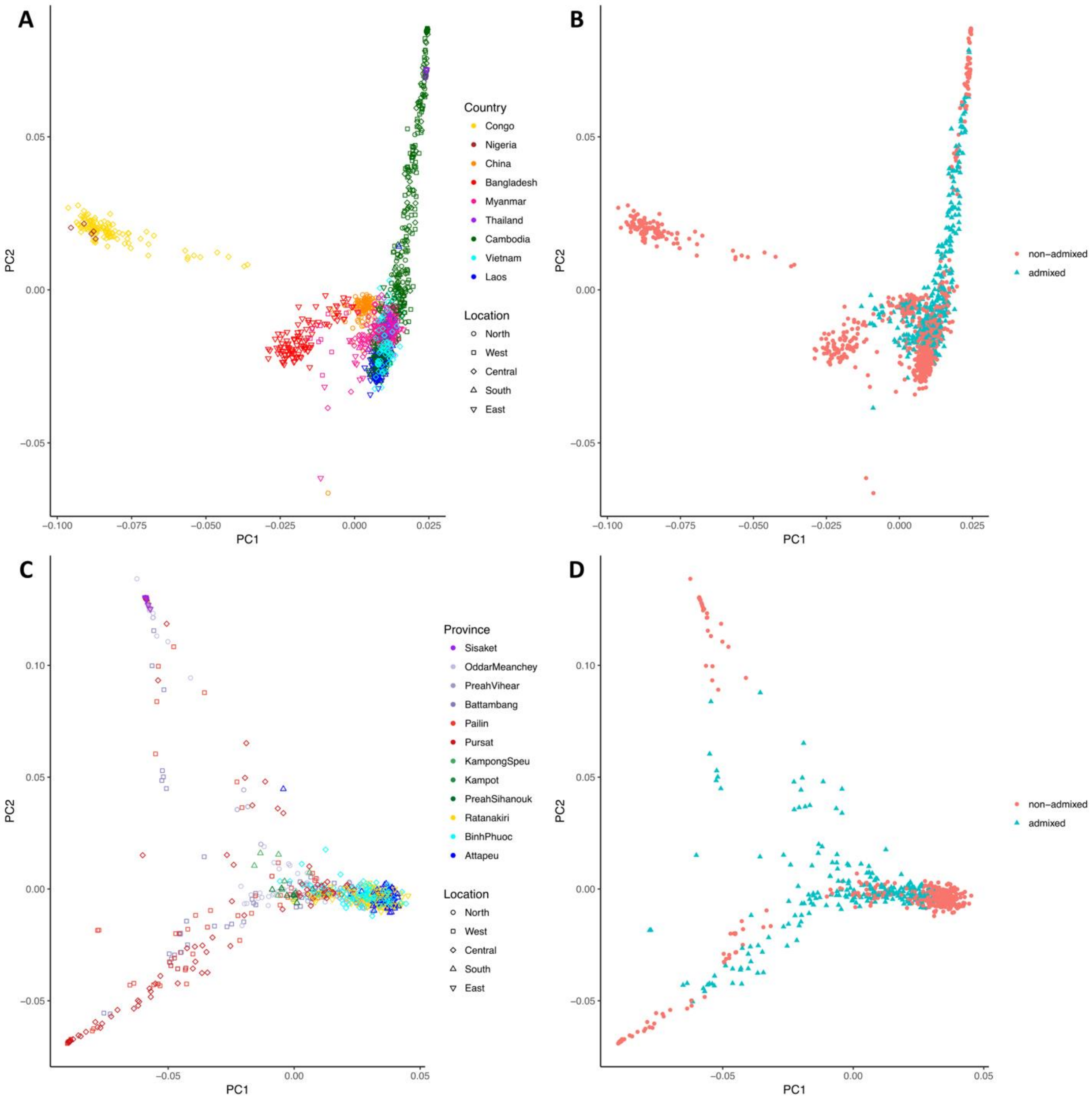

Supplementary Figure 5. Principal Component Analysis (PCA). PCA illustrates the first PC on the horizontal axis and the second PC on the vertical axis. The samples are colored by geographical location stratified by (A) country for all of Southeast Asia or (C) province within and around Cambodia with additional stratification by geographical position. Panels (B) and (D) illustrate the admixture status assigned to each sample in panels (A) and (C), respectively. PCA results corroborated admixture results with clear distinctions between African and Non-African populations. Isolates from China and Bangladesh clustered together while isolates from Thailand, Vietnam, and Laos illustrated less genetic diversity. Isolates from Cambodia spread along the 2nd PC show increased genetic diversity. Admixed samples are spread across the north-south cline observed along PC2.

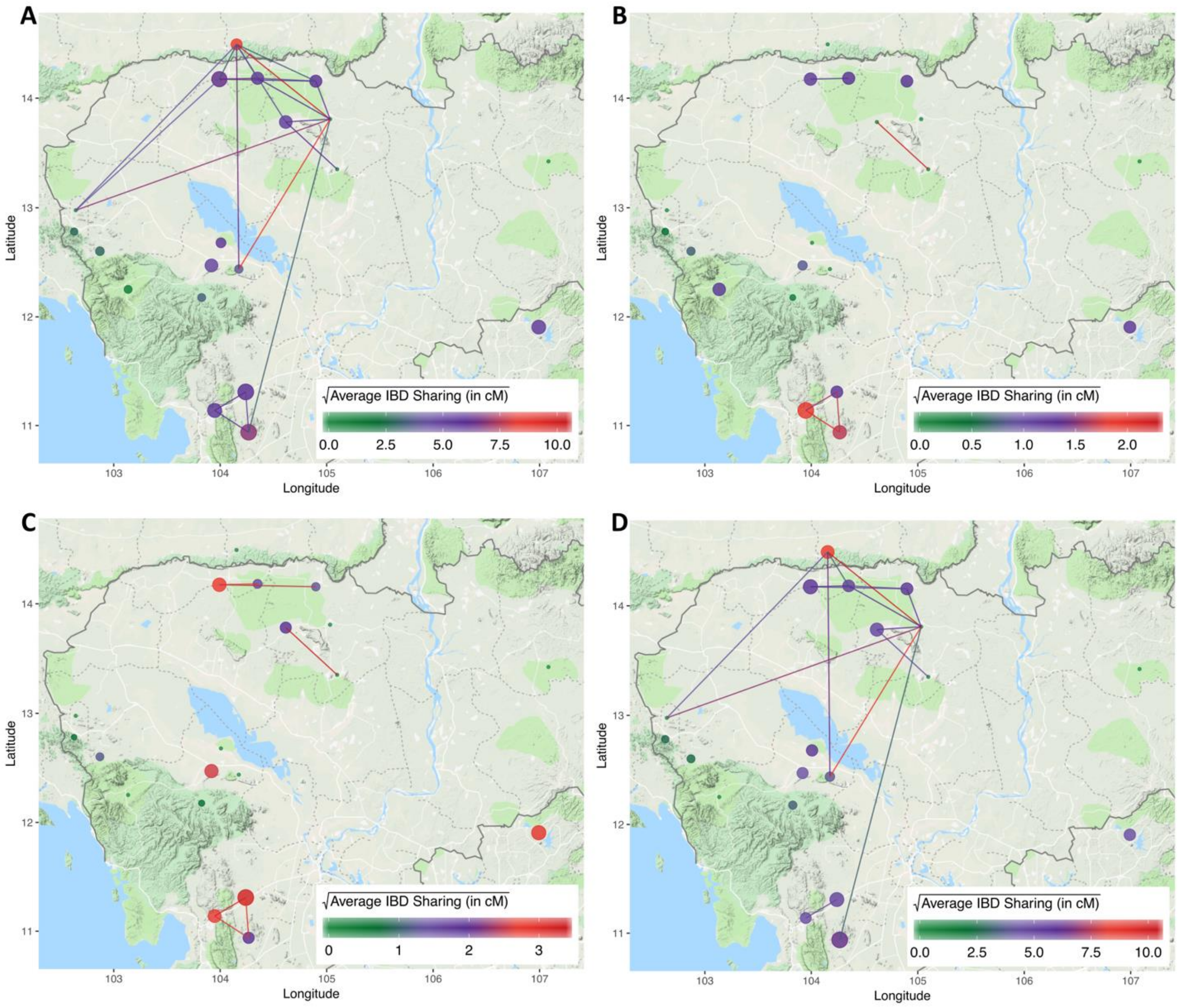

Supplementary Figure 6. Regional relatedness of K13 mutant parasites within and between districts. (A) All segments greater than 2cM, (B) 2cM – 15cM, (C) 15cM – 30cM, and (D) greater than 30cM. Sharing of larger IBD segments indicates migration that is more recent. Circles represent the average IBD sharing within a district while lines represent the average IBD sharing between two districts. The color indicates the magnitude of IBD sharing while the area of the circle represents the average number of segments shared. Only district-pairs with >15% isolate-pairs demonstrating IBD sharing are included. Map data: Google, 2018.

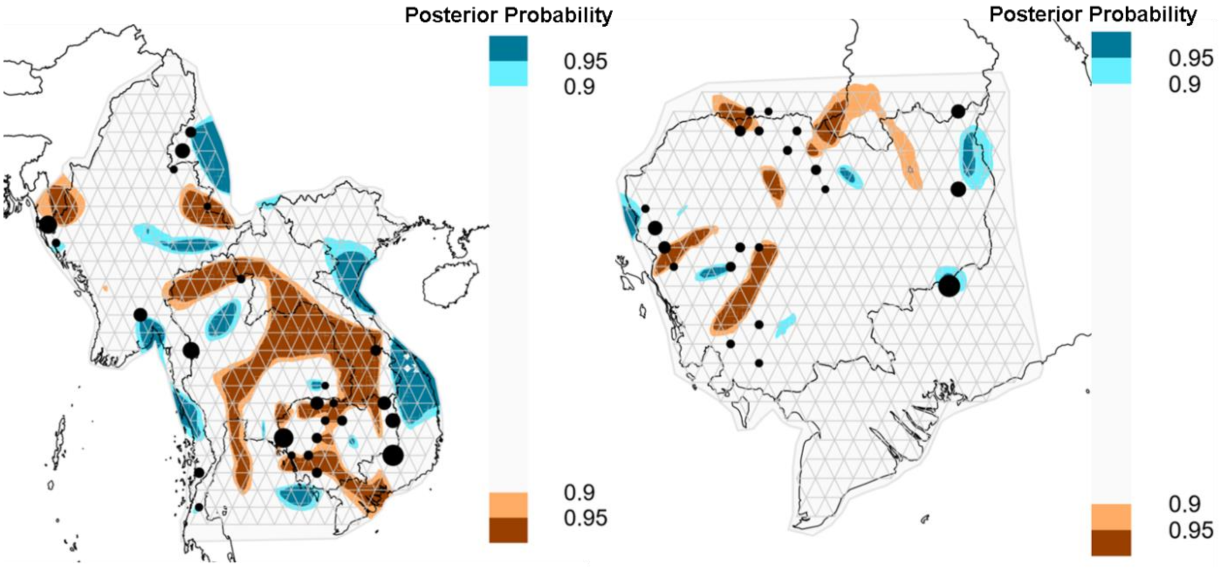

Supplementary Figure 7. Posterior probabilities of EEMS migration parameter estimates. EEMS posterior probability contours for Southeast Asia (left) and Cambodia (right). Each circle is a deme consisting of one or more districts. The area of the circle is proportional to the number of isolates included in the deme. Brown contours represent areas of low relative migration supported by posterior probabilities >0.90. Blue contours represent areas of high relative migration supported by posterior probabilities >0.90. Areas in gray are supported by posterior probabilities <0.90.

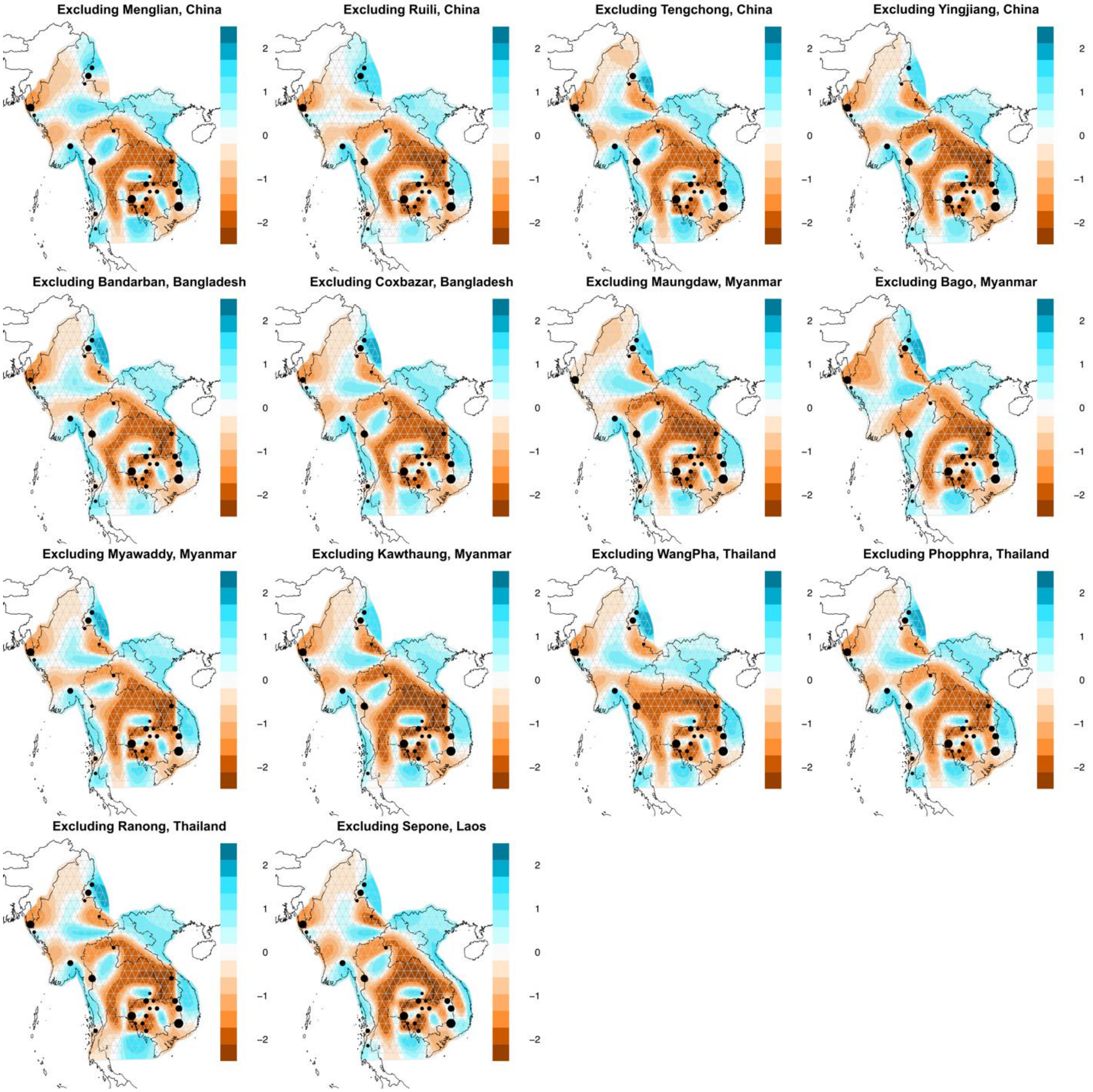

Supplementary Figure 8. EEMS sensitivity analysis in Southeast Asia. EEMS migration surface contours illustrating barriers to migration after iteratively excluding samples from a single district in Southeast Asia. Each circle is a deme consisting of one or more districts. The area of the circle is proportional to the number of isolates included in the deme. Brown contours indicate lower relative migration and blue contours indicate higher relative migration.

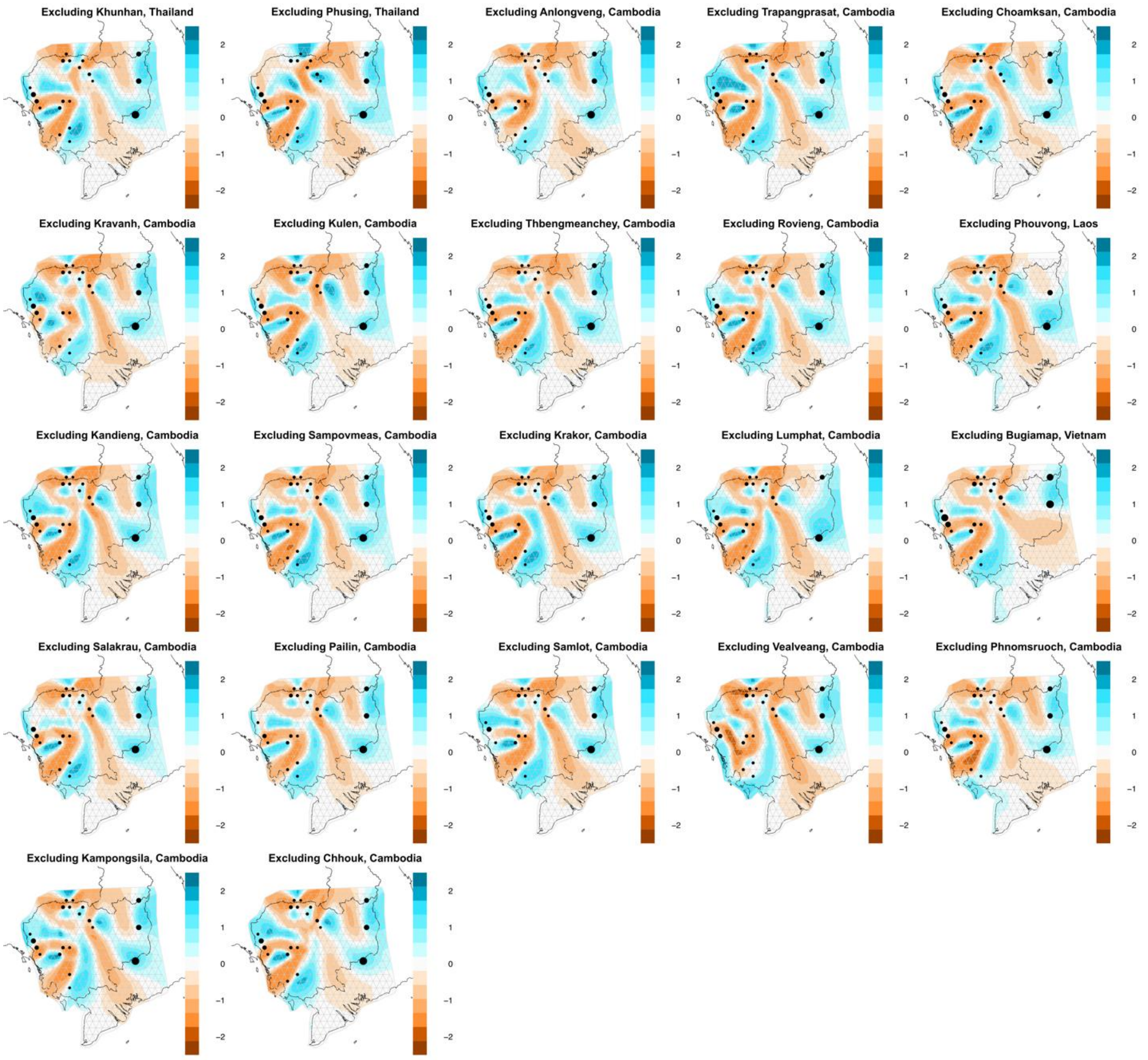

Supplementary Figure 9. EEMS sensitivity analysis in Cambodia. EEMS migration surface contours illustrating barriers to migration after iteratively excluding samples from a single district in Cambodia. Each circle is a deme consisting of 1 or more districts. The area of the circle is proportional to the number of isolates included in the deme. Brown contours indicate lower relative migration and blue contours indicate higher relative migration.

Supplementary Table 1. Location and average IBD sharing within districts.

Country	Province	District	Longitude	Latitude	Isolates	Average IBD Sharing (in cM)	# Isolates after excluding genetically similar isolates	Average IBD Sharing (in cM) after excluding genetically similar isolates
Bangladesh	Chittagong	Bandarban	92.37	21.81	61	1.8113	60	1.1750
		Coxs Bazar	92.07	21.49	46	3.4213	44	2.3509
Cambodia	Battambang	Kamrieng	102.57	13.12	2	NA	NA	NA
		Samlout	102.87	12.60	97	48.4310	45	9.6813
	KampongSpeu	Oral	104.06	11.74	3	84.7998	NA	NA
		Phnom Sruoch	104.24	11.31	18	47.3672	12	38.4303
		Samrong Tong	104.50	11.47	1	NA	NA	NA
	Kampot	Chhouk	104.27	10.94	15	67.4438	6	40.3167
	KohKong	Thma Baing	103.50	11.75	1	NA	NA	NA
	OddarMeanchey	Anlong Veng	103.99	14.17	48	59.9698	31	30.3557
		Samrong	103.61	14.25	1	NA	NA	NA
		Trapang Prasath	104.35	14.18	25	76.2241	14	34.6138
	Pailin	Pailin	102.63	12.78	138	46.6317	65	5.7528
		Sala Krau	102.64	12.98	14	80.9460	7	NA
	PreahSihanouk	Kampong Sila	103.95	11.14	11	39.9566	10	21.3176
	PreahVihear	Cheb	105.46	13.91	1	NA	NA	NA
		Chey Sen	105.35	13.58	2	NA	NA	NA
		Choam Ksan	104.90	14.15	28	148.2836	12	23.4221
		Kulen	104.62	13.78	26	69.4446	14	20.0781
		Roveing	105.10	13.35	6	128.5396	3	10.4097
		Sankumthmey	104.78	13.49	1	NA	NA	NA
		Thbeng Meanchey	105.03	13.81	79	102.7461	25	14.2678
	Pursat	Bakan	103.75	12.68	2	NA	NA	NA
		Kandieng	104.01	12.68	9	11.6793	5	9.4421
		Krakor	104.18	12.44	14	17.8843	8	16.0148
		Kravanh	103.49	12.18	36	21.3958	23	5.1955
		Sampov Meas	103.92	12.47	36	44.7104	16	16.1999
		Veal Veng	103.14	12.25	11	39.2087	7	2.5424
	Ratanakiri	Lumphat	107.08	13.42	102	20.5412	73	7.4625

Supplementary Table 1, Continued…

Country	Province	District	Longitude	Latitude	Isolates	Average IBD Sharing (in cM)	# Isolates after excluding genetically similar isolates	Average IBD Sharing (in cM) after excluding genetically similar isolates
China	Yunnan	Menglian	99.47	22.29	4	31.4918	4	31.4918
China	Yunnan	Ruili	97.81	24.04	7	9.9731	7	9.9731
China	Yunnan	Tengchong	98.51	25.26	45	15.8495	34	0.9406
China	Yunnan	Yingjiang	97.93	24.85	91	15.9801	74	3.9126
Laos	Attapeu	Phouvong	107.01	14.57	88	28.2729	61	6.4049
Laos	Attapeu	Saysetha	102.71	17.98	1	NA	NA	NA
Laos	Savannakhet	Sepone	106.34	16.76	40	17.2380	36	9.9934
Myanmar	Bago	Bago	96.59	17.73	87	23.3927	62	8.1529
Myanmar	Kayin	Myawaddy	98.53	16.54	122	40.4470	77	16.7014
Myanmar	Rakhine	Maungdaw	92.39	21.01	12	0.9376	12	0.9376
Myanmar	Tanintharyi	Kawthaung	98.77	10.99	51	53.9523	30	27.7112
Thailand	Nan	Wang Pha	100.75	19.13	13	2.3469	13	2.3469
Thailand	Ranong	Ranong	98.61	9.86	19	188.6042	10	5.4404
Thailand	Sisaket	Kantharalak	104.67	14.57	1	NA	NA	NA
Thailand	Sisaket	Khukhan	104.19	14.73	1	NA	NA	NA
Thailand	Sisaket	Khun Han	104.41	14.54	9	494.3826	4	54.5449
Thailand	Sisaket	Phu Sing	104.15	14.49	17	66.3505	13	56.0306
Thailand	Tak	Maeramat	98.59	17.10	3	55.4732	NA	NA
Thailand	Tak	Maesot	98.73	16.74	2	NA	NA	NA
Thailand	Tak	Phopphra	98.83	16.47	20	34.6918	13	11.2540
Vietnam	BinhPhuoc	Budang	107.22	11.78	2	NA	NA	NA
Vietnam	BinhPhuoc	Bu Gia Map	106.99	11.91	246	35.0462	133	4.9697
Vietnam	Soc Trang	Chauthanh	105.90	9.68	1	NA	NA	NA
Vietnam	BinhPhuoc	Phurieng	106.94	11.69	1	NA	NA	NA

Supplementary Table 2. Average IBD estimates within and between districts by country.

	Countries	>2cM	2cM - 15cM	15cM - 30cM	> 30cM
Within-District Sharing (Mean ± SD)	**China**	11.580 ± 13.797	0.985 ± 1.054	5.022 ± 6.469	5.573 ± 6.450
	**Bangladesh**	1.763 ± 0.832	0.450 ± 0.467	0.674 ± 0.457	0.639 ± 0.092
	**Myanmar**	13.376 ± 11.526	1.348 ± 1.255	4.338 ± 3.787	7.690 ± 6.491
	**Thailand**	25.923 ± 27.001	0.532 ± 0.711	1.507 ± 1.475	23.884 ± 28.767
	**Cambodia**	16.972 ± 12.317	0.987 ± 0.986	3.302 ± 3.478	12.684 ± 9.563
	**Vietnam**	4.970 ± NA	0.360 ± NA	1.525 ± NA	3.084 ± NA
	**Laos**	8.199 ± 2.537	0.581 ± 0.005	2.343 ± 0.197	5.275 ± 2.335
Between-District Sharing (Mean ± SD)	**China**	1.636 ± 2.406	0.190 ± 0.192	0.556 ± 0.652	0.890 ± 1.593
	**Bangladesh**	0.589 ± NA	0.231 ± NA	0.258 ± NA	0.099 ± NA
	**Myanmar**	0.589 ± 0.740	0.084 ± 0.097	0.223 ± 0.236	0.282 ± 0.417
	**Thailand**	8.339 ± 17.388	0.104 ± 0.291	0.293 ± 0.703	7.941 ± 17.505
	**Cambodia**	3.682 ± 5.322	0.208 ± 0.368	0.678 ±1.308	2.796 ± 3.992
	**Vietnam**	---	---	---	---
	**Laos**	0.046 ± NA	0.015 ± NA	0.032 ± NA	0.000 ± NA

Average IBD sharing within and between districts for each country shown in Figure 2

Supplementary Table 3. Directional migration inferred between non-admixed and admixed samples across districts

District 1 (D1)	District 2 (D2)	p-value	Average IBD Sharing (cM) (D1N_D2N)	Average IBD Sharing (cM) (D1N_D2A)	Average IBD Sharing (cM) (D2N_D1A)
**Anlong Veng**	**Kulen**	$<10^{-5}$	0.0000	3.2608	0.0000
**Anlong Veng**	**Sala Krau**	$<10^{-5}$	0.0000	3.6155	0.0000
**Bago**	**Ranong**	$<10^{-5}$	0.0000	1.7825	0.0000
**Kampong Sila**	**Kravanh**	$<10^{-5}$	0.0000	1.5613	0.0000
**Kampong Sila**	**Phnom Sruoch**	$<10^{-5}$	0.0000	18.0133	0.0000
**Kawthaung**	**Ranong**	$<10^{-5}$	0.0000	11.0543	0.0000
**Kravanh**	**Phnom Sruoch**	$<10^{-5}$	0.0000	1.4144	0.0000
**Kravanh**	**Sala Krau**	$<10^{-5}$	0.0000	11.6627	0.0000
**Pailin**	**Sala Krau**	$<10^{-5}$	0.0000	2.2660	0.0000
**Pailin**	**Veal Veng**	$<10^{-5}$	0.0000	1.9976	0.0000
**Phnom Sruoch**	**Samlout**	$<10^{-5}$	0.0000	0.0000	0.9519
**Rovieng**	**Thbeng Meanchey**	$<10^{-5}$	0.0000	0.0000	14.1607
**Sala Krau**	**Samlout**	$<10^{-5}$	0.0000	0.0000	3.7770
**Samlout**	**Sampov Meas**	$<10^{-5}$	3.0278	5.2322	0.0000
**Samlout**	**Veal Veng**	$<10^{-5}$	0.0000	2.0601	0.0000
**Sampov Meas**	**Veal Veng**	$<10^{-5}$	0.0000	3.5111	0.0000

Table denotes the average IBD sharing between non-admixed and admixed samples across districts. D1N, D2N, D1A, and D2A represent non-admixed and admixed isolates from district D1 and D2, respectively. All p-values were computed using a permutation test of 100,000 permutations.

Supplementary References

1 Chang, C. C. *et al.* Second-generation PLINK: rising to the challenge of larger and richer datasets. *Gigascience* **4**, 7 (2015).

2 Purcell, S. *et al.* PLINK: a tool set for whole-genome association and population-based linkage analyses. *Am J Hum Genet* **81**, 559-575 (2007).

3 Alexander, D. H., Novembre, J. & Lange, K. Fast model-based estimation of ancestry in unrelated individuals. *Genome Res* **19**, 1655-1664 (2009).

4 Alexander, D. H. & Lange, K. Enhancements to the ADMIXTURE algorithm for individual ancestry estimation. *BMC Bioinformatics* **12**, 246 (2011).

5 Manichaikul, A. *et al.* Robust relationship inference in genome-wide association studies. *Bioinformatics* **26**, 2867-2873 (2010).
