# Supplementary material for: Genomic structure and diversity of Plasmodium falciparum in Southeast Asia reveal recent parasite migration patterns
Source: Nat Commun. 2019 Jun 17;10:2665. doi: 10.1038/s41467-019-10121-3 (PMC6572796; doi:10.1038/s41467-019-10121-3)
Supplement: Supplementary file 2 — Reporting Summary [file 41467_2019_10121_MOESM2_ESM.pdf]

nature research

Corresponding author(s): Amol C Shetty
Timothy D O'Connor
Shannon Takala-Harrison

Last updated by author(s): Apr 1, 2019

Reporting Summary

Nature Research wishes to improve the reproducibility of the work that we publish. This form provides structure for consistency and transparency in reporting. For further information on Nature Research policies, see Authors & Referees and the Editorial Policy Checklist.

Statistics

For all statistical analyses, confirm that the following items are present in the figure legend, table legend, main text, or Methods section.

n/a	Confirmed	
☐	☒	The exact sample size (*n*) for each experimental group/condition, given as a discrete number and unit of measurement
☒	☐	A statement on whether measurements were taken from distinct samples or whether the same sample was measured repeatedly
☐	☒	The statistical test(s) used AND whether they are one- or two-sided<br>*Only common tests should be described solely by name; describe more complex techniques in the Methods section.*
☒	☐	A description of all covariates tested
☐	☒	A description of any assumptions or corrections, such as tests of normality and adjustment for multiple comparisons
☐	☒	A full description of the statistical parameters including central tendency (e.g. means) or other basic estimates (e.g. regression coefficient) AND variation (e.g. standard deviation) or associated estimates of uncertainty (e.g. confidence intervals)
☒	☐	For null hypothesis testing, the test statistic (e.g. *F*, *t*, *r*) with confidence intervals, effect sizes, degrees of freedom and *P* value noted<br>*Give P values as exact values whenever suitable.*
☐	☒	For Bayesian analysis, information on the choice of priors and Markov chain Monte Carlo settings
☐	☒	For hierarchical and complex designs, identification of the appropriate level for tests and full reporting of outcomes
☒	☐	Estimates of effect sizes (e.g. Cohen's *d*, Pearson's *r*), indicating how they were calculated

Our web collection on statistics for biologists contains articles on many of the points above.

Software and code

Policy information about availability of computer code

Data collection	N/A
Data analysis	PLINK (v. 1.9); BEAGLE (v.4.1); ADMIXTURE (v.1.3); R packages ggplot2, ggmap, and ecodist; Estimated Effective Migration Surfaces (EEMS); KING (v.1.4)

For manuscripts utilizing custom algorithms or software that are central to the research but not yet described in published literature, software must be made available to editors/reviewers. We strongly encourage code deposition in a community repository (e.g. GitHub). See the Nature Research guidelines for submitting code & software for further information.

Data

Policy information about availability of data

All manuscripts must include a data availability statement. This statement should provide the following information, where applicable:

- Accession codes, unique identifiers, or web links for publicly available datasets
- A list of figures that have associated raw data
- A description of any restrictions on data availability

Genotyping data are publicly available through the MalariaGEN website ([https://www.malariagen.net/data/p-falciparum-community-project-jan-2016-data-release]) or through the NIH Gene Expression Omnibus (Accession number GSE100704 [https://www.ncbi.nlm.nih.gov/geo/query/acc.cgi?acc=GSE100704]) and European Variation Archive (Accession number PRJEB28530 [https://www.ebi.ac.uk/ena/data/view/PRJEB28530]).

Field-specific reporting

Please select the one below that is the best fit for your research. If you are not sure, read the appropriate sections before making your selection.

☒ Life sciences ☐ Behavioural & social sciences ☐ Ecological, evolutionary & environmental sciences

For a reference copy of the document with all sections, see nature.com/documents/nr-reporting-summary-flat.pdf

Life sciences study design

All studies must disclose on these points even when the disclosure is negative.

Sample size	Sample size calculations were not performed. Sample size was based on the number of parasite isolates with high quality SNP data that were above the threshold for the proportion of missing SNP calls and for which district-level geographic coordinates were available. The sample sizes in this study were comparable or larger than than in other studies using similar approaches to estimate parasite population demography.
Data exclusions	Samples were excluded that were missing more than 10% of SNP calls. In analyses of regional relatedness, districts with <3 isolates were excluded to allow adequate sample size to make robust estimates while preserving the spatial density of the dataset. Analyses of regional relatedness were also performed both including and excluding highly genetically similar isolates to test the impact of these isolates on relatedness estimates.
Replication	The results have not been replicated in an independent dataset. However, we observed some similar migration patterns using two different approaches, with estimated effective migration surfaces (EEMS) showing similar patterns to those estimated based on IBD segments of small and intermediate lengths.
Randomization	This study was not a randomized controlled trial, and no randomization was used to group parasites. Parasite isolates were grouped by sampling district to determine connectivity between parasite populations in different geographic locations. To estimate asymmetrical migration, parasites were grouped into admixed and non-admixed groups.
Blinding	Investigators were not blinded to sampling district during the data analysis; however, IBD estimates were determined without regard to sampling district.

Reporting for specific materials, systems and methods

We require information from authors about some types of materials, experimental systems and methods used in many studies. Here, indicate whether each material, system or method listed is relevant to your study. If you are not sure if a list item applies to your research, read the appropriate section before selecting a response.

Materials & experimental systems

n/a	Involved in the study	
☒	☐	Antibodies
☒	☐	Eukaryotic cell lines
☒	☐	Palaeontology
☒	☐	Animals and other organisms
☐	☒	Human research participants
☒	☐	Clinical data

Methods

n/a	Involved in the study	
☒	☐	ChIP-seq
☒	☐	Flow cytometry
☒	☐	MRI-based neuroimaging

Human research participants

Policy information about studies involving human research participants

Population characteristics	No human samples were collected as part of this study. Samples were collected with informed consent from individuals with symptomatic malaria infection as part of previously conducted studies. Only the parasite genetics were of interest. Other than sampling district, human characteristics were not relevant to the data analysis.
Recruitment	No recruitment of human subjects was performed as part of this study. This study involved evaluation of malaria parasite genomic data generated from DNA extracted from archived samples from completed studies.
Ethics oversight	The University of Maryland School of Medicine Institutional Review Board approved of the parasite genotyping and analyses performed in this study.

Note that full information on the approval of the study protocol must also be provided in the manuscript.
